# Ascariasis, trichuriasis and fatal non-transfusion

**DOI:** 10.4322/acr.2021.314

**Published:** 2021-08-20

**Authors:** Larry Nichols, Joshua Curtis Bridgewater, Nicholas Brennan Wagner

**Affiliations:** 1 Mercer University School of Medicine, Department of Pathology and Clinical Science Education, Macon, GA, USA

**Keywords:** Ascariasis, Trichuriasis, Anemia, Blood Transfusion, Transfusion Reaction

## Abstract

Blood transfusion for chronic anemia can lead to acute or decompensated heart failure in patients who have fluid overload as part of their compensatory response and/or have intrinsic heart disease, and then it could be fatal in such clinical scenarios. This is the report of a case of profound chronic anemia in a young male patient, who was not transfused and then developed confusion followed by terminal cardiopulmonary arrest. Autopsy revealed severe trichuriasis to be the cause of the anemia, along with severe ascariasis, but minimal intrinsic brain disease. This supports the conclusion that anemia was the cause of the confusion, and the lesson that confusion may be a sign that the benefit of blood transfusion outweighs the risk in a patient with severe chronic anemia.

## CASE REPORT

A 29-year-old man from a rural area of Mexico suffered many years of physical abuse during his childhood. He had a seizure at age 24. Approximately 4 years later, he developed confusion, weakness, fatigue and diarrhea, so his mother brought him to the United States for medical care. Ten days following the onset of his symptoms and 8 days after his arrival in the United States, he was brought to an urban outpatient clinic in the Southwest United States. In the clinic at 14:00, his temperature was 36.7^o^ C, pulse rate 100 beats/minute, blood pressure 90/50 mm Hg, respiration rate 20 breaths/minute, hematocrit 16.3% (RR: 42-52%), and white blood cell (WBC) count 12,500/mm^3^ (RR: 4,500-11,000/mm^3^). An intravenous infusion of normal saline was started and the patient was sent to a large public hospital emergency department.

On arrival in the emergency department at 16:42, the patient’s pulse rate was 84 beats/minute, blood pressure 112/70 mm Hg and respiration rate 16 breaths/minute. He had a grade 2/6 systolic ejection murmur and yellow stool with gross blood in it. He was unable to cooperate for detailed interviewing or physical examination because of “altered mental status”. His hematocrit was 17.3%, hemoglobin 4.8 g/dL (RR: 14-18 g/dL), and WBC count 11,100/mm^3^. Chest x-ray showed patchy bilateral peripheral nodular infiltrates and pleural effusions.

The following morning, at 07:00, an order was written to transfuse 2 units of red blood cells over 1 hour each as soon as possible. The patient’s hemoglobin was 4.4 g/dL, hematocrit 14.9%, mean corpuscular volume (MCV 52.3 fL (RR: 80-100 fL), WBC count 15,500/mm^3^, bilirubin 0.3 mg/dL (RR: 0.1-1.2 mg/dL), alkaline phosphatase 150 U/L (RR: 35-110 U/L), alanine aminotransferase (ALT) 8 U/L (RR: 5-40 U/L), aspartate aminotransferase (AST) 12 U/L (RR: 5-40 U/L), lactate dehydrogenase (LDH) 285 U/L (RR: 120-300 U/L), albumin 2.3 g/dL (RR: 3.9-5 g/dL), blood urea nitrogen 11 mg/dL (RR: 10-20 mg/dL), creatinine 0.3 mg/dL (RR: 0.4-1.3 mg/dL), calcium 7.7 mg/dL (RR: 8.6-10.3 mg/dL), and creatine phosphokinase (CPK) 25 U/L (RR: 35-345 U/L). A hematology consultant obtained further blood tests showing hemoglobin 4.6 g/dL, hematocrit 16.5%, MCV 52.9 fL, WBC count 9,400/mm^3^ (58% segmented neutrophils, 1% bands, 14% lymphocytes, 4% monocytes, 22% eosinophils), folate 4.9 ng/mL (RR: 2.5-20 ng/mL), vitamin B12 419 pg/mL (RR: 250-1000) pg/mL), normal hemoglobin electrophoresis, and normal thyroid functions tests. Peripheral blood smear showed hypochromasia, microcytosis, teardrop cells, target cells, red blood cell fragments, anisocytosis, poikilocytosis and markedly increased platelets. The hematology consultant recommended parenteral iron instead of transfusion. The order for transfusion was rescinded, although the patient had already been premedicated for it. He was transferred from the emergency department to an inpatient unit where he passed two loose yellow foul-smelling stools with no blood in them.

The next morning, hospital day 3, the medicine resident who took over the patient's management noted confusion as a new problem. The medicine resident ordered a computed tomography scan of the head, which showed no abnormalities, and at 16:30, performed lumbar puncture. Opening pressure was 20 cm H_2_O. The cerebrospinal fluid was clear. Approximately 45 minutes following the lumbar puncture, the patient was found on the floor, unresponsive, with cyanosis, shallow respirations and fecal incontinence. He was given an ampule of 50% dextrose intravenously and supplemental oxygen at 6 L/minute. He became responsive, but remained confused. At 17:45, the patient ceased breathing and the cardiac arrest team was summoned. Despite full cardiopulmonary resuscitative efforts, the patient could not be revived.

## AUTOPSY FINDINGS

**Figure 1:**
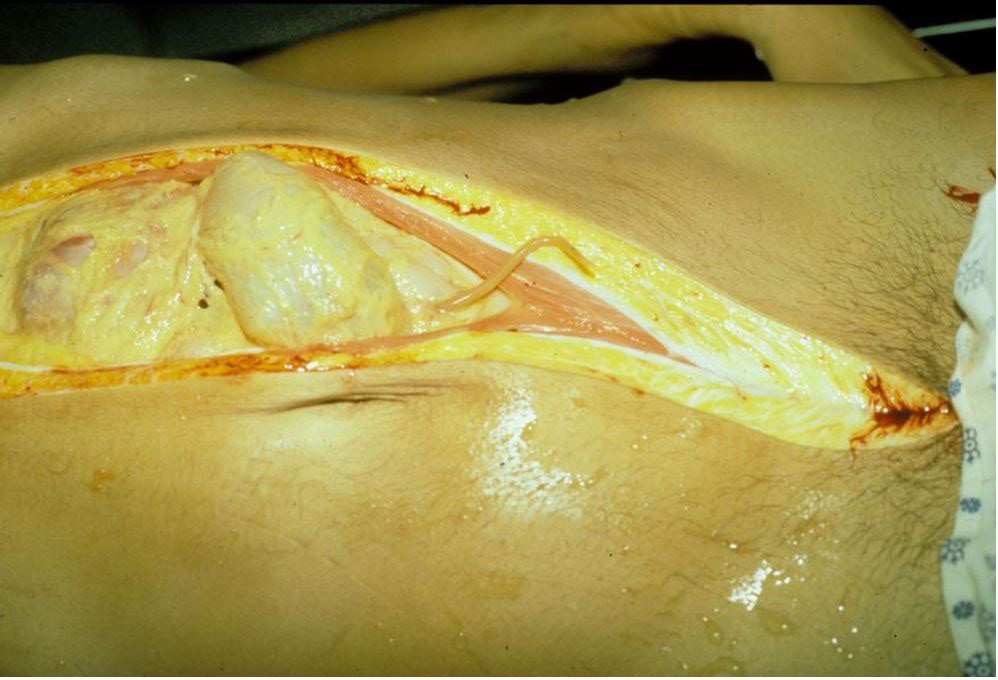
Ascaris emerging from inadvertently opened jejunum.

Waiting for the tardy autopsy assistant who usually made the primary incision, the pathologist became impatient and made the incision, inadvertently opening the jejunum through which a writhing Ascaris worm rapidly emerged and was photographed with a hand-held camera (Figure 1).

**Figure 2:**
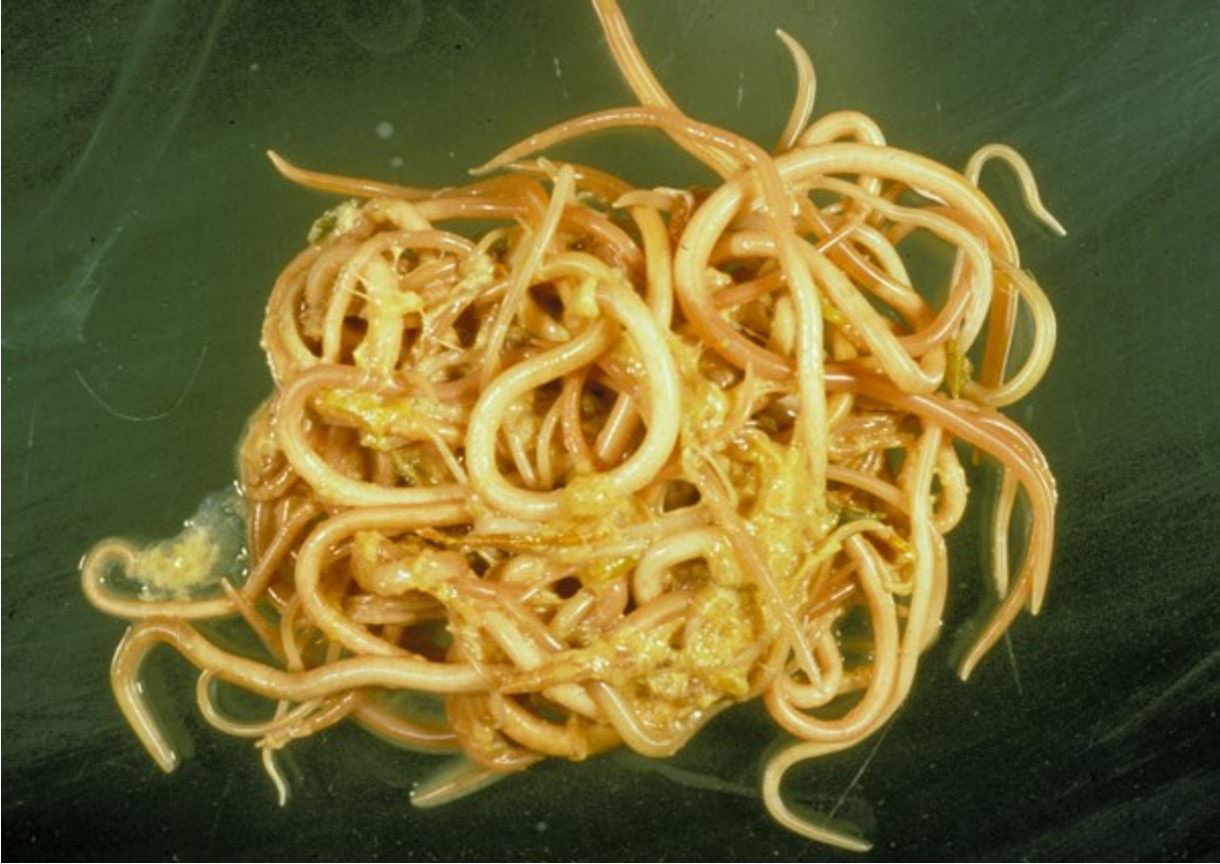
One of multiple aggregates of Ascaris removed from small bowel.

Further opening the small bowel revealed 50 to 100 Ascaris worms, up to 20 cm in length, which were removed, and one aggregate was taken to the specimen photography table and photographed (Figure 2).

**Figure 3:**
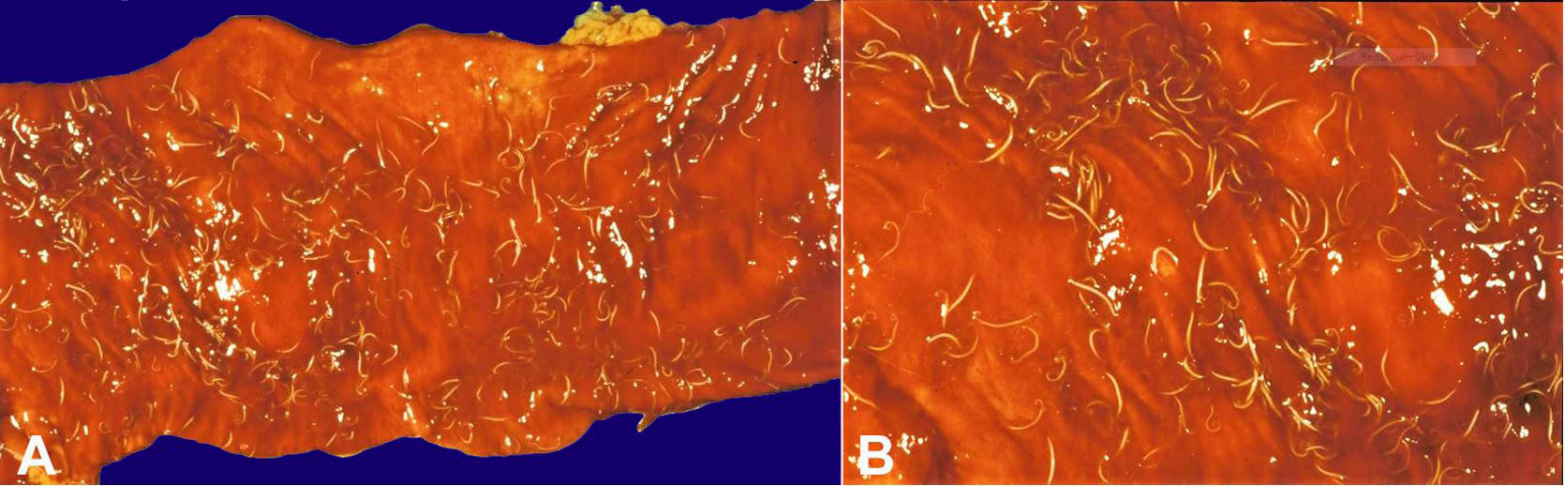
Gross view of the segment of hemorrhagic colon with trichuriasis; B - Closer view of the colonic trichuriasis.

At this point in the autopsy, the inexperienced pathologist proclaimed to the autopsy assistant that he had found the cause of the anemia. The autopsy assistant was from a region with a wide variety of helminthic diseases, and he informed the pathologist that the cause of the anemia had not been found because ascariasis does not cause anemia. Opening the colon, however, revealed extensive trichuriasis, with hundreds of Trichuris whipworms, up to 4 cm in length, embedded in erythematous and hemorrhagic colonic lining (Figures 3A, B). The autopsy assistant then informed the pathologist that he had found the cause of the anemia.

The body mass index was 19.7 kg/M^2^ (RR: 18.5-24.9 kg/M^2^). The heart weighed 360 g (RR: 221-386 g), with no chamber dilatation or any other abnormalities on gross or microscopic examination. The lungs had multiple firm tan nodules up to 3 cm in greatest dimension; microscopic examination revealed these to be granulomas, with central necrosis and peripheral organization and fibrosis. One granuloma contained a structure consistent with a necrotic ghost Ascaris. The brain showed mild edema and a microscopic gliomesenchymal cell nodule in the medulla, but no hemorrhages or other abnormalities. Stool culture was negative for Salmonella, Shigella, Yersinia, Arizona and Campylobacter species. Representative helminths were examined in the microbiology laboratory and identified as *Ascaris lumbricoides* and *Trichuris trichiura*.

## DISCUSSION

This patient had chronic anemia, which is a different species than acute anemia. One important difference is that patients with chronic anemia can have fluid overload as part of their compensatory response to chronic anemia. Intestinal parasites can cause chronic anemia. Ascariasis, which this patient had, is the most common helminthic infection in the world, afflicting more than 1.2 billion people.^1^ Between 5% and 12% of those with ascariasis have peripheral blood eosinophilia, which this patient had and which is more often associated with the lung migration phase of infection.^1^ Ascariasis does not typically cause anemia. Trichuriasis, which this patient had, is the second most common helminthic infection in the world, afflicting 795 million people.^2^ Severe trichuriasis can cause anemia. Each whipworm embeds into the colonic lining, with associated hemorrhage and daily blood loss of 0.005 mL per worm per day.^3^ The patient of this report had a heavy infection with hundreds of embedded helminths sufficient to account for his chronic anemia.

Transfusion for chronic anemia can lead to acute or decompensated heart failure in patients who have fluid overload as part of their compensatory response or have intrinsic heart disease.^4^ The syndrome of respiratory distress from pulmonary edema following blood transfusion is termed transfusion-associated circulatory overload (TACO). TACO is now recognized as the leading cause of transfusion-related morbidity and mortality, with an incidence ranging from 1% in low-risk populations to 12% in high-risk populations (patients with a history of coronary artery disease, heart failure or renal failure).^4^ Patients with TACO typically have hydrostatic pulmonary edema in association with elevated pulmonary venous pressure and systemic arterial pressure.^4^^,^ 5 In contrast, patients with transfusion-related acute lung injury (TRALI) have pulmonary edema from increased endothelial permeability, although there is some overlap in clinical manifestations and proposed pathophysiology.^4^ In addition to being dangerous in patients with chronic anemia, transfusion can be wasteful because most chronic anemia can be treated with measures such as oral iron supplementation or parenteral iron therapy if treatment is more urgent.^6^ Blood transfusion for chronic anemia can be fatal.^5^

Not transfusing blood for anemia can also be fatal. Specifically, in a study of patients hospitalized with myocardial injury (elevated troponin, not diagnosed as acute coronary syndrome) and anemia (hemoglobin < 8 g/dL, without overt bleeding), those not transfused blood were 2.27 times more likely to die.^7^ Similarly, a study of patients refusing blood transfusion found a 55% increase in mortality per 1 g/dL decrease in nadir hemoglobin below 8 g /dL.^8^ There is a critical hemoglobin concentration, where oxygen extraction is maximized and further decrease results in tissue ischemia, generally first manifest in brain dysfunction. The critical hemoglobin concentration at which this happens in humans without comorbidity is 5 g/dL.^8^ The patient of our report had hemoglobin below 5 g/dL and we believe this was most likely a case of fatal non-transfusion. Clearly, the decision to transfuse or not transfuse blood should not be taken lightly.^9^

In the case of this report, blood transfusion was ordered in the emergency department, but a hematology consultant recommended parenteral iron instead of transfusion, and the order for transfusion was rescinded. Parenteral iron therapy may risk worsening an infection by microbes competing for iron with the human they are infecting.^10^ This risk seems to be small.^11^ Active infection with sepsis from it would be a reason for withholding parenteral iron. Sepsis is life-threatening organ dysfunction caused by a dysregulated host response to infection. The clinical history suggests that the patient of our report had life-threatening organ dysfunction, but the autopsy showed chronic granulomatous inflammation rather than acute infection, so the organ dysfunction was more likely due to hypoxia than sepsis.

The morning after the order to transfuse the patient was rescinded, he was confused. The neuropathologist who signed out the brain commented that the mild cerebral edema at autopsy may have represented the effect of hypoxia, but noted that the patient died much too rapidly for any histopathologic evidence of hypoxic damage to be seen. Although the patient had a body mass index in the normal range, he was malnourished and it is reasonable to wonder if he might have had thiamine deficiency, which can cause disorientation among other manifestations of Wernicke encephalopathy.^12^ Thiamine is a vitamin with limited tissue storage, but folate is also a vitamin with limited tissue storage and the patient had a serum folate level in the normal range. Postmortem examination of the brain showed no hemorrhages or findings typical of Wernicke encephalopathy to account for the patient's altered mental status.

The diagnostic approach to the patient's confusion taken by the medicine resident displays an organ specific way of thinking. Computed tomography of the head and examination of cerebrospinal fluid are investigations of the central nervous system. In retrospect, knowing the autopsy results, one can see the patient's cerebral dysfunction as due to his anemia, inadequate oxygen delivery to the brain, rather than intrinsic brain disease. The approach taken in this case may have been due to a cognitive error of neglecting Occam's razor, seeking an additional diagnosis to explain the patient's confusion instead of realizing that it was a sign of the severe anemia already diagnosed. Alternatively, it may have been due to a cognitive error of seeking an organ specific diagnosis instead of realizing confusion was one of multiple signs and symptoms of anemia that the patient as a whole was suffering. Diagnostic errors of missed, wrong or delayed diagnoses occur in 8% to 15% of all hospital admissions in the United States and more than 75% of them are wholly or partially attributable to cognitive factors in clinician decision making.^13^ Clinician cognitive errors are prevalent everywhere that clinicians are human. This case is reported not to shame or blame, but rather to provide lessons in patient care from autopsy.

## CONCLUSION

This is the report of a case of profound chronic anemia due to trichuriasis in a patient also suffering malnutrition in association with ascariasis. Transfusion in circumstances like this can cause heart failure due to fluid overload. This patient was not transfused. He developed confusion and suffered a terminal cardiopulmonary arrest approximately 49 hours after hospitalization attributable to anemia. Confusion may be a sign that the benefit of transfusion outweighs the risk in a patient with severe chronic anemia.
